# Convolutional Neural Network-Based Compound Fingerprint Prediction for Metabolite Annotation

**DOI:** 10.3390/metabo12070605

**Published:** 2022-06-29

**Authors:** Shijinqiu Gao, Hoi Yan Katharine Chau, Kuijun Wang, Hongyu Ao, Rency S. Varghese, Habtom W. Ressom

**Affiliations:** Department of Oncology, Lombardi Comprehensive Cancer Center, Georgetown University Medical Center, Washington, DC 20057, USA; sg1506@georgetown.edu (S.G.); hc820@georgetown.edu (H.Y.K.C.); kw763@georgetown.edu (K.W.); ha623@georgetown.edu (H.A.); rsv4@georgetown.edu (R.S.V.)

**Keywords:** metabolite identification, deep learning, molecular fingerprint, metabolomics

## Abstract

Metabolite annotation has been a challenging issue especially in untargeted metabolomics studies by liquid chromatography coupled with mass spectrometry (LC-MS). This is in part due to the limitations of publicly available spectral libraries, which consist of tandem mass spectrometry (MS/MS) data acquired from just a fraction of known metabolites. Machine learning provides the opportunity to predict molecular fingerprints based on MS/MS data. The predicted molecular fingerprints can then be used to help rank putative metabolite IDs obtained by using either the precursor mass or the formula of the unknown metabolite. This method is particularly useful to help annotate metabolites whose corresponding MS/MS spectra are missing or cannot be matched with those in accessible spectral libraries. We investigated a convolutional neural network (CNN) for molecular fingerprint prediction based on data acquired by MS/MS. We used more than 680,000 MS/MS spectra obtained from the MoNA repository and NIST 20, representing about 36,000 compounds for training and testing our CNN model. The trained CNN model is implemented as a python package, MetFID. The package is available on GitHub for users to enter their MS/MS spectra and corresponding putative metabolite IDs to obtain ranked lists of metabolites. Better performance is achieved by MetFID in ranking putative metabolite IDs using the CASMI 2016 benchmark dataset compared to two other machine learning-based tools (CSI:FingerID and ChemDistiller).

## 1. Introduction

Liquid-chromatography coupled with mass spectrometry (LC-MS) is one of the most common technologies used to evaluate the levels of small molecule metabolites in biological samples. However, metabolite annotation continues to be a major challenge in LC-MS for untargeted metabolomics studies. Whereas spectral matching of experimental tandem mass spectrometry (MS/MS) data against those in spectral libraries is the gold standard for metabolite annotation, the limitations of spectral libraries create a bottleneck in untargeted metabolomics studies. This is because the MS/MS spectra in publicly accessible spectral libraries cover only a fraction of known compounds [1,2,3]. In addition to the limited number of spectra acquired by analysis of reference compounds, the difference in instrument methods between those in spectral libraries and those acquired by users seeking to annotate unknown metabolites poses a significant challenge.

Machine learning (ML) has been used for metabolite annotation based on experimental MS/MS spectra [4]. The approach involves designing a machine learning model to teach (train) the computer to predict fingerprints from training spectra and use the trained model to curate samples based on the learned fingerprints. A fingerprint of a compound indicates the presence or absence of a particular property or substructure of the compound, which is represented in binary digits [5]. The training involves multiple steps including MS/MS data pre-processing, feature selection, and model parameter section to capture the unknown relationships between molecular fingerprints of compounds and experimental MS/MS spectra associated with the compounds [6]. After the training is completed, the trained model is used to predict a fingerprint based on MS/MS spectrum. The metabolite candidates corresponding to the MS/MS spectrum are then retrieved from a compound database(s) based on its precursor mass. The corresponding candidates are ranked on the basis of the similarities between the fingerprints of the candidates and the predicted one.

A large family of linear and nonlinear models including logistic regression (LR), support vector machine (SVM), and artificial neural networks (ANNs), etc. have been investigated for compound fingerprint prediction and metabolite annotation. For example, SIMPLE is a hybrid machine learning model for predicting MS/MS peak relationships [7]. CSI:FingerID applies SVM for metabolite annotation from MS/MS spectral data [8]. Deep neural network is implemented in a model SIRIUS 4 for isotope patterns detection from MS/MS spectral data [9]. While selecting the architecture of the machine learning model is critical, selecting the features and factors that affect the relationship between a compound and its MS/MS spectrum is also important. For example, factors that may vary the spectral pattern include analytical and experimental settings such as collision energy, ionization mode, MS resolution, adduct, and the type of MS being used [10,11].

We previously applied multi-layer perceptron (MLP) to predict compound fingerprints and rank metabolite candidates obtained from compound databases based on mass values or formulae of the unknown compounds [12]. We reported a comparison among different machine learning models on annotating metabolites from NIST 17 and CASMI 2016 datasets. In this paper, we investigated the use of a convolutional neural network (CNN) for compound fingerprint prediction. The architecture of the CNN is more complex (containing convolutional layer, pooling layer, nonlinear activation function, fully connected layer, and dropout layer) than MLP (that contains input layer, hidden layer and output layer) [13]. One of the common characteristics of MLP and CNN is that the number of total parameters can grow remarkably high as each node is fully connected with every node in the receding layer. This may cause redundancy, overfitting, and inefficiency when the number of parameters and data size increases. CNN has a pooling layer in between each convolutional layer and the main purpose of these pooling layers is to reduce data dimension and lower the amount of calculation [13]. This results in a higher efficiency during the training process as well as avoiding overfitting problems [13]. CNN has two major advantages over MLP. First, CNN has a parameter sharing mechanism. Since CNN and MLP all have the same filter for different regions, they share the same set of parameters. A filter is used to detect a feature that is likely to appear in more than one place. Parameter sharing mechanism, so that the number of parameters of our network greatly reduced. In this way, we can train a better model with fewer parameters and effectively avoid overfitting. Therefore, CNN is more promising for accurately mapping the relationships between MS/MS spectra and molecular fingerprints [14].

The CNN has a rising application in metabolomics including peak recognition [14]. For instance, Zhang et al. trained DeepSpectra, a CNN module for pattern recognition from raw near infrared spectral data [15]. Kim et al. applied CNN and developed SMART-Miner for identifying 2D NMR peaks from a mixture sample for metabolite identification [16]. Fedorova et al. applied and found one-dimensional CNN predicts the most accurate retention time in reversed-phase liquid chromatography [17]. DeepEI, a CNN-based method, was designed to predict molecular fingerprints from the electron ionization mass spectrometry (EI-MS) spectrum [18]. DEEP Picker applies eight hidden convolutional layers for peak picking and spectral deconvolution of two-dimensional NMR spectra purposes [19].

In this paper, we evaluated multiple machine learning models in mapping the relationships between molecular fingerprints and MS/MS spectra. In addition to cross-validation based on training and testing data sets from MoNA and NIST 20, we used models trained by combining all the MoNA and NIST 20 MS/MS datasets for evaluation via the CASMI 2016 benchmark dataset. Due to its superior performance compared to other machine learning models, we chose to implement the trained CNN into a python package, MetFID. We compared the performance of MetFID against CSI:FingerID and ChemDistiller in ranking metabolite candidates using the CASMI 2016 dataset. Instead of using the CASMI candidates for ranking, each tool was subjected to generate its own candidates based on precursor *m/z* of the MS/MS spectra in the CASMI 2016 benchmark datasets. We also investigated the use of three inputs (collision energy, mode, and resolution) in addition to the peak intensities. Furthermore, we explored the use of eight separate CNN models for each permutation of three instrument methods instead of one CNN model. We observed the use of eight separate models leads to more promising performance than a single model in ranking the candidates.

## 2. Results

### 2.1. Evaluation of the CNN’s Performance in Metabolite Annotation

Table 1 shows the evaluation results of the CNN model compared with other models including LR, SLP, SVM, and MLP in predicting fingerprints based on five-fold cross validation on training MS/MS spectra acquired from 29,588 compounds. We used multi-output regression for linear model. Also, for the Multi-Layer Perceptron (MLP) and SVM, the input transformed spectra consisted of 1174 bins after the binning process. We also removed bins that consisted of all 0′s across all the spectra in the training set to reduce the dimension of the input. The removal did not cause any loss of information from those bins since all the training sets were 0′s in those bins and there was no information for classification. For the CNN model, we did not remove the bins with 0′s. Table 1 presents the F1 score and Tanimoto similarity score between the true fingerprint and the predicted fingerprint for the validation dataset using the five-fold cross validation. The cross-validation method was also used when we tuned the hyperparameter for the CNN model.

We use top-*k* ranking performance to evaluate the CNN model’s performance in metabolite annotation. The ranking accuracy in Table 1 refers to the percentage of testing cases in which the correct metabolite appears in the top-*k* of the ranked candidate list. The results show that the CNN model successfully ranked the correct identification in more than 40% of the cases. We also evaluated the CNN assuming that the formula of the unknown compounds is known; in this case, CNN successfully ranked the correct identification in 50% of the cases. For these datasets, we observed that the use of formula information helps shrink the average length of candidate lists from a large number to about 6 to 12 candidates. 

We compared the performance of CNN against SLP, SVM, and MLP in annotating analytes in the CASMI 2016 benchmark datasets. Each model was trained with 35,878 spectra extracted from the NIST 20 and MoNA libraries. Table 2 presents the annotation results. While all four models have great performance, the SLP model has the lowest F1 and Tanimoto values. Both SVM and MLP yielded lower F1 and Tanimoto scores than CNN. All models show better performance on formula-based ranking than mass-based ranking.

### 2.2. Evaluation of Single vs. Multiple CNNs

Based on the top-*k* ranking, we observed that the performance of eight separate models trained by splitting the spectra based on instrument methods (collision energy, resolution, and ionization mode) is slightly better (2–5%) than the single CNN model trained with or without the additional three inputs. The performance of the trained model by adding entries to the input vector is similar to that of a model trained without the additional inputs. We used 80% of the spectra as the training set and the remaining spectra excluding the training (structurally disconnected) compounds for testing. Results are presented in Table S1 in the Supplementary Materials. The results indicate that the Eight CNNs overall perform best among the three approaches. Furthermore, we trained the Eight CNNs by combining the training and testing MoNA and NIST 20 spectra. The top-*k* performance of the trained Eight CNNs were then compared with the One CNN model by using the CASMI 2016 dataset for testing. As shown in Table S2 of the Supplementary Materials, the One CNN model performed better.

### 2.3. Evaluation of MetFID’s Performance Compared to Other Tools

To compare with ChemDistiller and CSI:FingerID, we packaged into MetFID the One CNN model that was trained using spectra obtained from NIST 20 and MoNA and selecting those that are structurally-disjoint to the CASMI 2016 benchmark dataset. We used the CASMI 2016 testing set to compare MetFID vs. CSI:FingerID and ChemDistiller. Details of the tools and all the parameters are listed in Table S3 of the Supplementary Materials. We tested ChemDistiller and CSI:FingerID that were trained with their own testing spectra. ChemDistiller was compared with MetFID using the mass-based method and CSI:FingerID was compared with the formula-based method. We did not use our library testing to compare these three models because NIST 20 spectra were involved in the training of the current version of CSI:FingerID. For performance evaluation, we used the CASMI 2016 benchmark dataset that consists of 208 peak lists from 188 substances, 127 peak lists were acquired in the positive mode 81 in the negative mode. A list of candidate metabolites is provided along with SMILES, InChI, and InChIKey for each peak list. 

For CSI:FingerID, we performed metabolite annotation by using SIRIUS 4, software for analyzing metabolites from tandem mass spectrometry(MS) data which combines isotope pattern analysis in MS with fragment pattern analysis in MS/MS and uses CSI:FingerID as a web service to search in molecular structure database. We accessed this tool and provided information with processed peak lists, exact mass, and precursor adduct of each of the challenge (peak list) in the CASMI 2016 dataset. The 208 peak lists were entered into SIRIUS 4 as a benchmark data set for three parts computation, SIRIUS, ZODIAC, and CSI:FingerID. SIRIUS identifies the molecular formula for the measured precursor ions and annotates the spectrum by providing the molecular formula for each fragment peak. ZODIAC improves the ranking of the formula candidates provided by SIRIUS. CSI:FingerID identifies the structure of a compound by searching a molecular structure database. After computing, we obtained a formula-based top-*k* rank which included candidate’s InChIKey for each peak list. Then, we compared the candidate’s InChIKey and the compound true InChIKey to perform top-*k* ranking of the candidates. When we used the candidate list from the CASMI 2016 data, CSI:FingerID was able to rank the true candidate at the top consistently for all 208 peak lists. We assumed this is because the CASMI 2016 spectra may have been included in the training of the SVM model used by CSI:FingerID. To evaluate the performance of the CNN model with previously unseen MS/MS data, we ensured that all training spectra are structurally disjointed compared to the compounds corresponding to the CASMI 2016 dataset. When we used the candidates of CSI:FingerID, 21 compounds were not annotated in the ranked result or not considered as true candidates. Table 3 presents the ranking results calculated in two ways: (1) excluding the CASMI 2016 spectra for which the true compound is missing in the candidate list; and (2) considering all CASMI 2016 spectra in the testing set regardless of the presence of the true compound in the candidate list. As shown in Table 3, MetFID has a better performance than CSI:FingerID.

ChemDistiller is a freely available metabolite annotation tool developed using a machine learning approach. Since there is no user interface available for this tool, the source code (in Python) of ChemDistiller was downloaded and directly operated through Anaconda. Two pre-trained support vector machine-learning (SVM) models are available in the ChemDistiller package, and each model was trained with fingerprints and fragmentation patterns. A linear SVM model and a radial SVM model were pre-trained with MassBank and NIST14 spectra through fingerprints (FingerScorer) and fragmentation patterns (FragScorer) [20]. There are also multiple databases made available by the authors of ChemDistiller. We were able to use BMDB, ChEBI, DrugBank, EcoCycMINE, HMDB, KEGGMINE, MassBank, YMDBMINE, and TestDB as the candidate databases of the run. As a user, the precursor mass (*m/z*), precursor ion (adduct), exact mass, database source, formula, InChI, ionization mode, and MS/MS peak data could be provided to the tool. With each CASMI 2016 sample, precursor mass, precursor ion, ionization mode, and MS/MS peak data, which is the lowest requirement for ChemDistiller to be able to give a result, were provided for annotation. ChemDistiller is capable of running sample data in bulk, so the same candidate lists were used on all 208 CASMI 2016 samples with each SVM model, and the same set of information was provided to the tool. After successfully running all the samples, the final annotation results are reported in an HTML file and a .txt file. The annotation results of each sample are ranked according to a score calculated by the tool. Information like formula, mass, charge, isotope, SMILES, InChI, and InChIKey is used to evaluate the performance of the tool.

As shown in Table 3, MetFID yielded far better performance in top-*k* ranking of putative metabolite IDs compared to CSI:FingerID and ChemDistiller. For example, assuming the formula of the unknown compounds are known, the correct compound name was ranked first in 71% of the cases by MetFID vs. 67% by CSI:FingerID. The correct compound name was ranked in the top three in 88% of the cases by MetFID vs. 71% by CSI:FingerID. When assuming the formula is unknown, MetFID outperformed the linear SVM model in ChemDistiller in all rankings as shown in Table 3. The radial SVM model of ChemDistiller achieved less top-*k* ranking performance than the linear model as illustrated Table S4 in the Supplementary Materials. In addition to testing MetFID using candidates generated by itself, we evaluated its top-*k* ranking performance based on candidates provided by CASMI 2016 as shown in Table S5 in the Supplementary Materials. 

## 3. Discussion

It has been a long debate and search about which machine learning model is capable of carrying more accurate compound fingerprint prediction for metabolite annotation. Here, we introduce a convolutional neural network (CNN) for fingerprint prediction. Briefly, we used 650,553 MS/MS spectra obtained from the MoNA repository and NIST 20 representing 35,878 compounds for training and testing the CNN model. About 80% of the MS/MS spectra representing 29,588 compounds were used for training. The trained model was evaluated using the remaining MS/MS spectra representing 6290 compounds that are structurally disjointed to those used for training. Compared to other machine learning methods, including support vector machine (SVM) and multi-layer perceptron (MLP), the proposed CNN model achieved better performance in predicting compound fingerprints and ranking metabolite putative metabolite IDs. Also, we observed very promising results in the ability of the CNN model for fingerprint prediction and ranking metabolite candidates. We demonstrated that the use of the CNN model leads to improved fingerprint prediction accuracy and ranking of metabolite candidates compared to the previously used MLP and SVM models. The trained CNN model is implemented as a python package, MetFID. The package is available on GitHub for users to enter their MS/MS spectra and putative metabolite IDs to obtain ranked lists of metabolites.

The performance of the trained MetFID in ranking putative metabolite IDs was compared with two other machine learning-based tools (CSI:FingerID and ChemDistiller) using the CASMI 2016 benchmark dataset, which consists of 208 MS/MS spectral representing 188 compounds. Although the level of improvement achieved by MetFID compared to ChemDistiller and CSI:FingerID was small, we anticipate promising potential for further improvement as more MS/MS data from reference compounds become available for training the CNN model. Also, we observed improved performance when multiple CNN models are used by segmenting the spectra according to the instrument type/settings (e.g., collision energy, instrument resolution, and positive/negative ionization mode). With the availability of more MS/MS spectra from different platforms, we will investigate the use of multiple CNN models.

Future work will focus on updating the CNN architecture to improve its prediction performance. Specifically, we will investigate the use of 2D CNN for compound fingerprint prediction. Our initial implementation of a 2D CNN by converting the input vector representing a spectrum into a data matrix did not yield better performance than the 1D CNN. Since a 2D CNN is more complex structurally than 1D CNN and typically used for image classification, further investigation is needed to adopt it for fingerprint prediction. Furthermore, we will investigate to better understand the relationships between molecular fingerprints with tandem MS fragmentation patterns and the potential use of other properties to calculate compound fingerprints such as the extended-connectivity fingerprints (ECFPs) that have been recently developed to capture molecular features relevant to molecular activity [9,21]. Finally, we will investigate feature selection methods to determine the most relevant *m/z* bins, instrument settings, and fingerprints prior to training a deep learning model.

## 4. Materials and Methods

### 4.1. Workflow Overview

**Figure 1:**
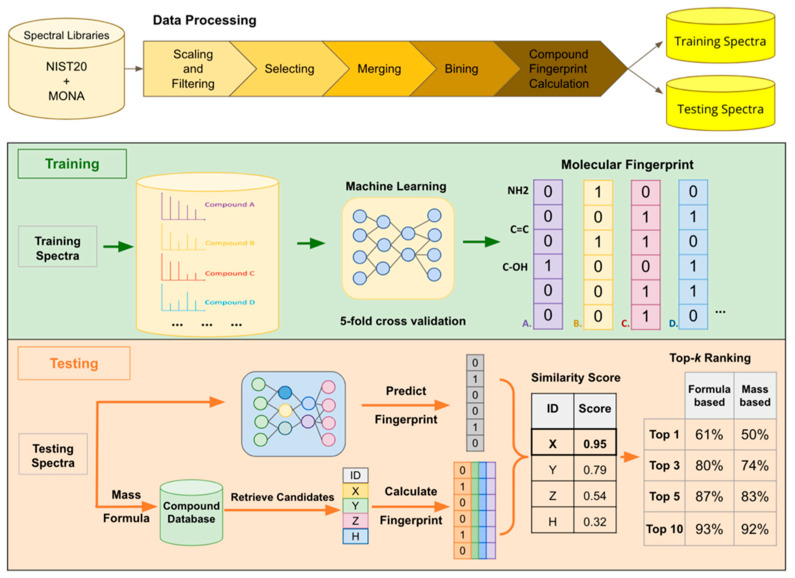
Machine learning-based compound fingerprint prediction for metabolite annotation.

The workflow consists of three phases: spectral processing, model training, and performance evaluation, as depicted in Figure 1. In the spectral processing phase, the MS/MS data collected from spectral libraries are transformed into vectors of intensity values based on predefined *m/z* bins. The vectors are amenable to be used as inputs to a computational model. In the training phase, the model is trained to map the relationship between MS/MS spectra and compound fingerprints. In the performance evaluation phase, previously unseen MS/MS spectra are converted into vectors and used as inputs to the trained model to predict the fingerprints of the unknown metabolites. In addition, candidate metabolite IDs are retrieved from compound databases based on the precursor *m/z*’s corresponding to the MS/MS spectra. The list of candidates can be narrowed if the molecular formulae for the unknown metabolites are known or calculated. For each candidate, the molecular fingerprint is calculated using OpenBabel [5]. The similarity between the molecular fingerprint predicted by CNN and the calculated molecular fingerprint is used to rank the candidates. Specifically, Tanimoto similarity score is used for ranking. If the true metabolite ID is known, the Tanimoto score, F1 score, and top-*k* ranking accuracy are used to evaluate the performance of the CNN model in molecular fingerprint prediction and metabolite annotation. In the subsequent sections, dataset preparation and the steps of the workflow are discussed. 

### 4.2. Data Processing

Spectral libraries. We downloaded MS/MS spectra acquired by LC-MS/MS from libraries available in the MoNA repository including Vaniya/Fiehn Natural Products Library, GNPS, RIKEN PlaSMA, MassBank, HMDB, MetaboBASE, Pathogen Box, Fiehn HILIC, etc. In addition, we obtained the NIST 20 library from one of NIST’s MS/MS library distributors. In the remainder of this paper, we will refer to MS/MS data downloaded from MoNA as MoNA spectra and those downloaded from NIST 20 as NIST 20 spectra. For each spectrum extracted from MoNA and NIST 20, 16 factors were extracted from the raw file, including InChIKey, SMILES, spectrum ID, source, ionization mode, adduct, precursor mass, exact mass, instrument type, instrument, collision energy, mass accuracy, library, external IDs, peak masses, and peak intensity. The peak list we obtained from each spectrum was processed and transformed into a vector for use as input to a machine learning model. 

Scaling and filtering. Min-Max scaling was applied to the peak list each spectrum. We scaled the peak intensities such that all intensity values lay between 0 and 100. Spectra that consisted of fewer than five peaks with relative intensity above 2% were removed. Also, we removed peaks whose *m/z* values were larger than the precursor mass. 

Selecting. To make the training MS/MS data as homogeneous as possible, we selected from the NIST 20 spectra those acquired via instrument types such as Orbitrap, QqQ, Q-TOF, or ion trap (IT). We obtained 79,404 LC-MS/MS spectra in positive mode and 32,269 LC-MS/MS spectra in negative mode from MoNA. From NIST 20, we obtained 401,985 LC-MS/MS spectra in positive mode and 136,895 LC-MS/MS spectra in negative mode from NIST 20. Thus, we considered a total of 650,553 MS/MS spectra representing 35,878 compounds. 

Merging. Peak lists were merged into one if they were acquired from the same compound using InChIKey as compound identifier. An additional filtering was applied for selecting the spectra if the mass falls within a mass range of 100 to 1010 Da. 

Binning. We binned the *m/z* range of each MS/MS spectrum into prespecified bins, which are continuous integer *m/z* values, and calculated the accumulated intensities within each bin as feature values. In machine learning models, each bin is considered as individual unordered bins. A bin that consists of all 0′s across all the spectra were removed in the train the training set for dimension reduction depends on the model selection. For example, the bins that consisted of all 0′s were not removed since the bins should be consecutive when running the convolution. This binning method has been applied previously [22,23].

Compound fingerprint calculation. Molecular fingerprints of all compounds in the training set were determined by Openbabel [5]. Specifically, the MACCS, FP3, and FP4 fingerprints were mined and assembled into a vector consisting of 528 binary items.

### 4.3. Training

**Figure 2:**
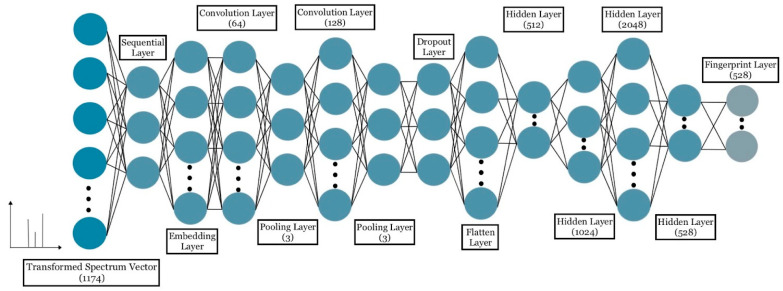
Architecture of CNN.

Following the data processing, we trained a CNN model to learn the relationship between spectral patterns and compound fingerprints. Figure 2 depicts the architecture of the one-dimensional CNN (1D CNN) we trained using the Keras Python package on the back end of TensorFlow. As shown in Figure 2, a CNN model consists of 12 layers (a Sequential layer, an Embedding layer, two Convolution1D layers, two MaxPooling1D layers, a Dropout layer, a Flatten layer, and four Hidden layers) following the input layer. Rectified Linear Unit (ReLU) was used as an activation function. We chose this activation function to alleviate the large amount of computation and the disappearance of the gradient when the error gradient is sought by the back propagation. Also, ReLU makes the output of some neurons equal to 0, which results in the sparsity of the network and reduces the interdependence of parameters, thus alleviating the occurrence of overfitting problem. The model uses multiple nodes in the output layer to predict all entries of fingerprint vector simultaneously without the need to build a separate model for each entry. The fingerprint prediction is a dichotomy problem, and the predicted output of the model is a vector with zeroes and ones. We therefore used Sigmoid as the final layer classification function of the CNN model and the binary cross-entropy as the loss function, rather than the usual combination of Softmax activation and the classification cross-entropy loss function used for single-tag tasks. For training, we used ‘Adam’ optimizer and ‘binary accuracy’ metrics. 

### 4.4. Testing 

We used MetaboQuest to retrieve candidates along with compound names, InChIKeys, formulae, etc. from compound databases such as HMDB, MMCD, MELIN, LIPID MAPS, KEGG based on the precursor *m/z*’s of MS/MS spectra with 20 ppm tolerance and considering different adduct forms. For each compound in the candidate list, we calculated the fingerprint. The candidate list was shortened if the formula of the compound is known by excluding those with different formulae. In practical cases, where the formulae are unknown for experimental MS/MS spectra, putative compound formulae can be calculated based on *m/z* values of precursor ions using tools such as SIRIUS [9].

After the list of candidates and the corresponding fingerprints were obtained, Equation (1) was used to calculate the Tanimoto similarity score between the predicted fingerprint of unknown compounds and the fingerprint of each candidate. The similarity scores that range from 0 to 1 were used to rank the candidates.

(1) $$\mathrm{Tanimoto similarity score}=\frac{\mathrm{TP}}{\mathrm{TP}+\mathrm{FP}+\mathrm{FN}}$$

To evaluate the performance of the trained CNN, we used previously unseen compounds in a structurally disjointed setting, which means the compounds in the training set cannot have the same first part of InChIKey as the compounds in the testing set. The MS/MS spectra from these compounds were processed in the same way as the training dataset, with the exception of merging. Thus, the performance of the CNN is evaluated using individual MS/MS spectra (without merging those acquired by different collision energies) in terms of its ability to perform both fingerprint prediction and metabolite annotation.

To achieve a balance between precision and recall rate, F1 and Tanimoto similarity scores were used to measure the fingerprint prediction performances of the CNN. Let TN, TP, FN, and FP represent the number of true negative, true positive, false negative, and false positive respectively. The precision equation was TP/P, and the recall equation was TP/(TP + FN). Equation (2) was used to calculate the F1 scores. In calculating the Tanimoto similarity score, we removed the part where the predicted fingerprint and the true fingerprint were both zeros, due to the significance of imbalance between the number of zeros and ones in the fingerprint.

(2) $$F1\;\mathrm{score}=\frac{2\;*\;\mathrm{precision}\;*\;\mathrm{recall}}{\mathrm{precision}\;+\;\mathrm{recall}}$$

To assess the performance of CNN in metabolite annotation, we ranked the retrieved candidates based on Tanimoto similarity scores calculated using Equation (1). In order to access the metabolite annotation accuracy of the top-*k* predicted candidates, we searched the true compound InChIKey in the first *k* (e.g., 1, 3, 5, and 10) predicted lists. Finally, we compared the performance of CNN against other models such as Logistic Regression, Single-Layer Perceptron (SLP), Support Vector Machine (SVM), and Multi-Layer Perceptron (MLP) models based on F1 score, Tanimoto similarity score, and top-*k* ranking results. Of the 35,878 compounds, 29,588 compounds (~80%) were used for training the CNN and other machine learning models. The remaining 6290 compounds, which were selected to be structure-disjoint to the training set, were used for independent testing.

### 4.5. Single vs. Multiple CNN Models

**Figure 3:**
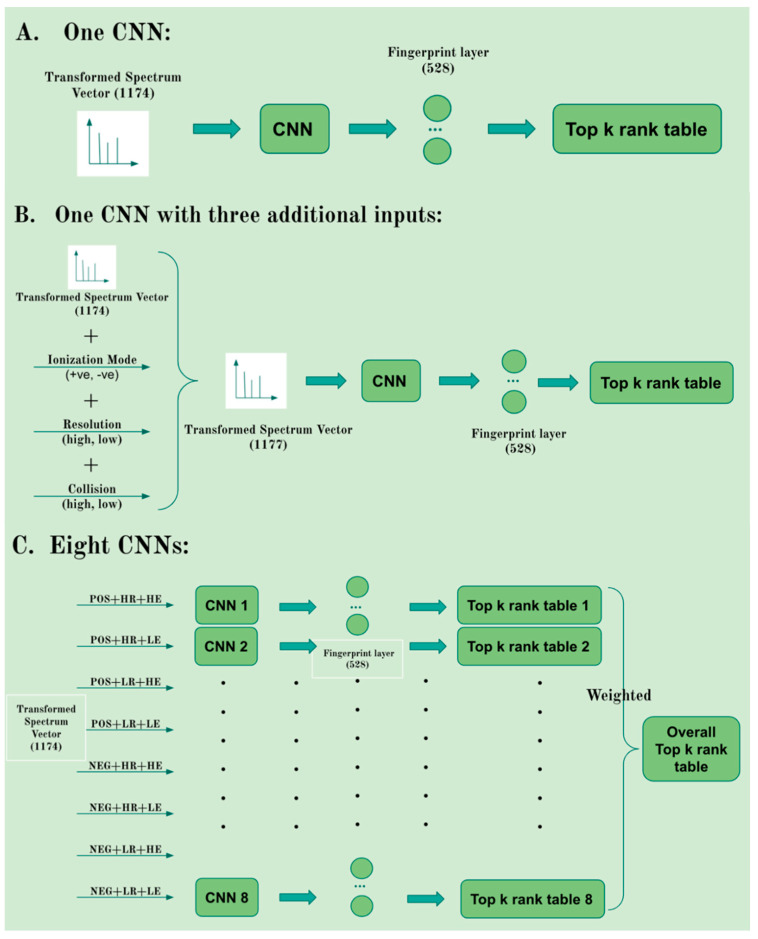
Three different strategies of the CNN model. (**A**) One CNN model with only ion intensity values as input. (**B**) One CNN model with ion intensity values and three additional inputs. (**C**) Eight CNN models each trained with a subset of the MS/MS spectra.

We investigated the potential advantage of training CNN model using different subsets of data on three features: collision energy, instrument types (LR-low resolution or HR-high resolution), and adduct forms (positive or negative). First, we divided the raw data into six separate datasets for low energy < 30 eV, high energy ≥ 30 eV, low resolution, high resolution, positive adducts, and negative adducts. Specifically, we used the spectra from the NIST 20 library, which provides collision energy information, at least one spectrum corresponding to <30 eV collision energy, and one spectrum corresponding to ≥30 eV collision energy, and for the MoNA library which contains more than 900 collision energy formats, we considered < 30 eV for low energy and ≥30 eV for high energy to extract the exact collision energy information from the MoNA library. We split the compounds with low resolution and high resolution from the NIST 20 library and MoNA library according to the instrument type. Instrument types such as Orbitrap and Q-TOF were considered as high resolution, while Ion Trap and QQQ were considered as low resolution. Figure 3 depicts three approaches: (A) one CNN model as explained in Section 4.2 representing the MS/MS spectrum with a vector consisting of 1174 bins that provide peak intensity values; (B) one CNN model representing the MS/MS spectrum with a vector consisting of 1174 bins and additional three binary entries to designate the spectrum as low/high energy, low/high resolution, positive/negative mode; and (C) eight CNN models each trained with a subset of datasets corresponding to a specific combination of energy (low/high), resolution (low/high), and ionization mode (positive/negative). The first two approaches take advantage of training the CNN with large sample size without the need to split the MS/MS spectra according to instrument type/setting.

## Figures and Tables

**Table 1:** Comparison of CNN with other machine learning models based on F1 score, Tanimoto similarity score, and the top-*k* ranking of the candidates selected by mass-based and formula-based search against compound databases for 29,588 training and 6290 testing compounds. The result of the best performing model under each category is shown in bold.

	**LR**		**SLP**		**SVM**		**MLP**		**CNN**	
**F1**	61%		59%		66%		67%		**71%**	
**Tanimoto**	45%		43%		52%		53%		** 58% **	
	**Mass-Based**					**Formula-Based**				
**Rank**	**LR** ^1^	**SLP**	**SVM**	**MLP**	**CNN**	**LR**	**SLP**	**SVM**	**MLP**	**CNN**
**Top 1**	32%	35%	39%	40%	**43%**	45%	47%	49%	48%	**50%**
**Top 3**	59%	61%	66%	66%	**69%**	68%	68%	70%	**71%**	**71%**
**Top 5**	71%	72%	75%	75%	**77%**	75%	75%	77%	77%	**78%**
**Top 10**	81%	82%	83%	82%	**84%**	81%	**82%**	**82%**	81%	**82%**

^1^ LR: Logistic Regression; SLP: Single-Layer Perceptron; SVM: Support Vector Machine; MLP: Multilayer Perceptron; CNN: Convolutional Neural Network.

**Table 2:** Performance comparison of SLP, MLP, SVM, and CNN models that were trained using MS/MS spectra acquired from NIST 20 and MoNA and tested on the CASMI 2016 dataset. F1 score, Tanimoto similarity score, and top-*k* ranking of metabolite candidates are calculated for mass-based and formula-based approaches. The result of the best performing model under each category is shown in bold.

**Dataset**	**CASMI 2016**							
**Libraries**	**NIST 20 + MoNA**							
	**SLP**		**SVM**		**MLP**		**CNN**	
**F1**	49%		52%		53%		56%	
**Tanimoto**	33%		36%		38%		41%	
	** Mass-Based **				**Formula-Based**			
**Rank**	**SLP**	**SVM**	**MLP**	**CNN**	**SLP**	**SVM**	**MLP**	**CNN**
**Top 1**	35%	32%	48%	**52%**	60%	59%	68%	**71%**
**Top 3**	61%	59%	71%	**76%**	81%	81%	87%	**88%**
**Top 5**	78%	75%	83%	**87%**	88%	86%	90%	**91%**
**Top 10**	93%	89%	93%	**95%**	94%	92%	94%	**95%**

**Table 3:** Performance comparison of ChemDistiller and CSI:FingerID with MetFID using the CASMI 2016 dataset as a testing dataset. The percentage that is outside the parenthesis includes the unannotated peak lists in the ranked result, which means 208 spectra in total. The percentages inside parenthesis are calculated by excluding the peak lists whose candidate lists do not include the true compounds, in order to account for the situation when the target compound cannot be found by searching against compound databases. The result of the best performing tool under each category is shown in bold.

CASMI 2016 Testing				
	Mass-Based		Formula-Based	
Rank	ChemDistiller	MetFID	CSI:FingerID	MetFID
**Top 1**	34% (44%)	**52%** (**53%**)	67% (73%)	**71%** (**74%**)
**Top 3**	47% (59%)	** 76% ** (**76%**)	71% (78%)	**88%** (**91%**)
**Top 5**	58% (73%)	** 87% ** (**87%**)	72% (79%)	**91%** (**94%**)
**Top 10**	63% (80%)	** 95% ** (**96%**)	72% (79%)	**95%** (**99%**)

## Data Availability

MetFID package is publicly available for download on GitHub (https://github.com/ressomlab/MetFID). The CASMI 2016 benchmark data set were obtained from Critical Assessment of Small Molecule Identification (http://www.casmi-contest.org/2016, accessed on 24 May 2021). MoNA data were downloaded from MassBank of North America (https://mona.fiehnlab.ucdavis.edu, accessed on 25 March 2021); and NIST 20 data were acquired from an official distributor (accessed on 20 March 2020).

## Supplementary Materials

The following supporting information can be downloaded at https://www.mdpi.com/article/10.3390/metabo12070605/s1, Table S1: Comparison of F1 scores, Tanimoto scores and top-*k* ranking results of the three CNN strategies, Table S2: Comparison of top-*k* ranking results between the Eight CNN model and the One CNN model, Table S3: Details of ChemDistiller, CSI:FingerID and MetFID, Table S4: Top-*k* ranking results of ChemDistiller with its Linear SVM and Radial SVM models on CASMI 2016 benchmark dataset, and Table S5: Top-*k* ranking results of MetFID with CASMI 2016 benchmark dataset and candidates.
