# Supplementary material for: Convolutional Neural Network-Based Compound Fingerprint Prediction for Metabolite Annotation
Source: Metabolites. 2022 Jun 29;12(7):605. doi: 10.3390/metabo12070605 (PMC9316655; doi:10.3390/metabo12070605)
Supplement: Supplementary file 1 [file metabolites-12-00605-s001.zip › metabolites-1758364-supplementary.pdf]

Supplementary Materials

Table S1. F1 score, Tanimoto similarity scores, and top-*k* ranking performance results of CNN models trained using three different strategies illustrated in Figure 3. The result of the best performing model under each category is shown in bold.

	One CNN	One CNN (With additional three inputs)	Eight CNNs			
**F1**	**68%**	67%	66%			
**Tanimoto**	**54%**	52%	51%			
	**Mass-Based**			**Formula-Based**		
**Rank**	**One CNN**	**One CNN (With additional three inputs)**	**Eight CNNs**	**One CNN**	**One CNN (With additional three inputs)**	**Eight CNNs**
**Top 1**	43%	42%	**44%**	52%	51%	**55%**
**Top 3**	71%	71%	**74%**	76%	75%	**79%**
**Top 5**	81%	80%	**85%**	82%	82%	**87%**
**Top 10**	89%	89%	**94%**	87%	88%	**93%**

Table S2. Comparison of top-*k* ranking performance results between the Eight CNNs model and One CNN model with one CNN model. Both models were trained with MoNA and NIST 20 data and tested with CASMI 2016 dataset. The percentages in parenthesis represent results when peak lists with candidates that miss the true compound are excluded from the calculation of the percentage. The result of the best performing model under each category is shown in bold.

	One CNN		Eight CNNs	
**F1**	**56%**		51%	
**Tanimoto**	**41%**		36%	
	**Mass-Based**		**Formula-Based**	
**Rank**	**One CNN**	**Eight CNNs**	**One CNN**	**Eight CNNs**
**Top 1**	**52%** (**53%**)	45% (46%)	**71%** (**74%**)	70% (72%)
**Top 3**	**76%** (**76%**)	70% (72%)	**88%** (**91%**)	87% (90%)
**Top 5**	**87%** (**87%**)	83% (85%)	**91%** (**94%**)	**91%** (**94%**)
**Top 10**	95% (96%)	94% (97%)	95% (**99%**)	**96%** (**99%**)

Table S3. Details of ChemDistiller, CSI:FingerID, and MetFID.

Tool	ChemDistiller	CSI:FingerID	MetFID
**Version Number**	ChemDistiller v0.1.	SIRUIS 4.8.2	Version 1.0.0
**Parameters**	Linear SVM and Radial SVM (Downloaded from https://www.mediafire.com/folder/v4lb8s2nns9c6/SVMs, replace current SVM in "SVMs" folder for each run)<br><br>Databases: BMDB, ChEBI, DrugBank, EcoCycMINE, HMDB, KEGGMINE, MassBank, YMDBMINE and TestDB (Downloaded from https://www.mediafire.com/folder/v5l4380gqbvie/DBs, files were put in DBs folder)<br><br>Precursor_ion: [M+H]+ for positive CASMI 2016 spectra samples; [M-H]- for negative CASMI 2016 spectra samples<br><br>Number of processors to use: 1<br><br>Max. results for each query: 100 candidates<br><br>m/z value tolerance: 20 ppm<br><br>*All other parameters were default.<br><br>Download page: https://bitbucket.org/iAnalytica/chemdistillerpython/downloads/	Adduct: $[M+H]^+$ for positive CASMI 2016 spectra samples; $[M-H]^-$ for negative CASMI 2016 spectra samples<br><br>Fallback Adducts: same as the Adduct setting listed above<br><br>Instrument type Q Exactive Plus Orbitrap (the instrument used by CASMI 2016)<br><br>Databases: all databases included in this version of CSI:FingerID<br><br>MS2 MassDev(ppm): 20 ppm<br><br>*All other parameters were default.<br><br>Homepage and Download page: https://bio.informatik.uni-jena.de/software/sirius/	1D-CNN model (1174 input vectors, 2 convolutional layers: one with 64 nodes, one with 128 nodes; 2 pooling layers after each convolutional layer; following by 4 hidden layers: 512 nodes, 1024 nodes, 2048 nodes, 528 nodes sequentially)<br><br>Databases MoNA (downloaded from: https://mona.fiehnlab.ucdavis.edu/downloads) and NIST 20<br><br>Testing data MoNA and NIST 20 for cross evaluation, CASMI 2016 for performance test<br><br>Mass tolerance: 20 ppm<br><br>*All other parameters were default.<br><br>Download page: https://github.com/ressomlab/MetFID/

Table S4. Top-*k* ranking performance result of ChemDistiller with its pre-trained linear SVM and radial SVM models on 208 CASMI 2016 peak lists. The percentages in parenthesis represent results when peak lists with candidates that miss the true compound are excluded from the calculation of the percentage. The result of the best performing model under each category is shown in bold.

Rank	Linear SVM	Radial SVM
**Top 1**	**34% (44%)**	28% (36%)
**Top 3**	**47% (59%)**	42% (54%)
**Top 5**	**58% (73%)**	50% (63%)
**Top 10**	**63% (80%)**	59% (74%)
**> 10**	**16% (20%)**	21% (26%)
**Not ranked**	**21%**	**21%**

Table S5. Top-*k* rank performance of MetFID on the CASMI 2016 benchmark dataset when the candidates provided by CASMI were used. The result of the best performing model under each category is shown in bold.

CASMI 2016 Testing + Library		
**Rank**	**Mass-Based**	**Formula-Based**
**Top 1**	45%	**54%**
**Top 3**	72%	**75%**
**Top 5**	77%	**80%**
**Top 10**	79%	**86%**
